# Assessment of Prevalence, Associations ,Knowledge, and Practices about Diabetic Foot Disease in a Tertiary Care Hospital in Colombo, Sri Lanka

**DOI:** 10.1155/2020/4504627

**Published:** 2020-11-26

**Authors:** V. T. S. Kaluarachchi, D. U. S. Bulugahapitiya, M. H. Arambewela, M. D. Jayasooriya, C. H. De Silva, P. H. Premanayaka, A. Dayananda

**Affiliations:** ^1^Diabetes and Endocrinology Unit, Colombo South Teaching Hospital, Kalubowila, Colombo, Sri Lanka; ^2^Department of Physiology, Faculty of Medical Sciences, University of Sri Jayewardenepura, Diabetes and Endocrinology Unit, Colombo South Teaching Hospital, Kalubowila, Colombo, Sri Lanka

## Abstract

**Background:**

One in five adults in Sri Lanka has either diabetes or prediabetes, and one-third of those with diabetes are undiagnosed. Diabetic foot is a debilitating condition affecting up to 50% of patients with both type 1 and type 2 diabetes. The risk of nontraumatic lower limb amputations is 15 times higher in diabetic patients when compared with nondiabetics. Patient education about correct foot care practices is the cornerstone of prevention of diabetic foot disease.

**Objective:**

To assess the prevalence of diabetic foot disease, knowledge, and practices about diabetic foot care among diabetic patients.

**Methods:**

334 patients attending the diabetic clinic in Colombo South Teaching Hospital were recruited according to the inclusion and exclusion criteria. Data were collected using 3 questionnaires, and they were filled using the foot examination findings, patients' medical records, and direct interviewing of the patients.

**Results:**

The mean age of the patients included in the study was 58.23 ± 10.65 years while the median duration of diabetes was 10.54 ± 7.32 years. 34.1% patients had peripheral neuropathy, and 29.5% had peripheral vascular disease. Diabetic foot disease according to the WHO definition was present only in 23 (6.9%) patients. There was a significant association between peripheral neuropathy and current or past foot ulcer which took more than 2 weeks to heal (*p* < 0.05). Knowledge about foot care was less among the studied population, and it was associated with poor foot care practices. Presence of diabetic foot and current or past foot ulcer which took more than 2 weeks to heal were significantly associated with the foot care knowledge and practices (*p* < 0.05)

**Conclusion:**

Improvement of patients' knowledge about foot care and their practices have a significant impact on the reduction of diabetic foot disease.

## 1. Introduction

Being a noncommunicable disease, diabetes accounts for a significant proportion of morbidity and mortality worldwide. Diabetes mellitus is a group of physiological dysfunctions characterized by hyperglycemia resulting directly from insulin resistance or inadequate insulin secretion leading to macrovascular and microvascular complications with time. Estimated community prevalence of diabetes in Sri Lankan urban population was 27.6% according to the recent study done in Sri Lanka, and this is an exponential rise over the last 3 decades [1]. This is higher than the urban prevalence of 18% shown by Katulanda et al. 12 years ago [2].

The International Working Group on the Diabetic Foot (IWGDF) has defined the diabetic foot as infection, ulceration, or destruction of tissues of the foot of a person with currently or previously diagnosed diabetes mellitus, usually accompanied by neuropathy and/or peripheral arterial disease (PAD in the lower extremity [3]. Diabetic foot is a debilitating complication of diabetes due to its effects on peripheral nerves and peripheral vessels, affecting up to 50% of patients with both type 1 and 2 diabetes. Peripheral nerve disease leads to loss of sensation predisposing to injuries while peripheral vascular disease affects the blood supply leading to poor healing. This complication significantly affects the quality of life of the patient. Furthermore, it represents at least 12–15% of the overall cost associated with diabetes and in developing countries, this is up to 40% [4].

When considering the Sri Lankan setup, the main cause of lower extremity amputations in Sri Lanka is diabetic foot ulcer according to Ubayawansa et al. [5]. They have found that diabetes was contributing to more than 50% of amputations directly or indirectly.

Many risk factors were identified as risk factors for diabetic foot such as long duration of diabetes, poor diabetic control, foot deformities, and presence of other diabetic complications [6]. According to a research done in Sri Lanka in 1996, one-third of all non-insulin-dependent diabetes patients attending the clinic had a risk of foot ulceration. 30.6% of patients had neuropathy according to the criteria used. 10.2% had a foot ulcer, a history of foot ulceration, or a lower extremity amputation due to neuropathic ulceration. 4.8% had a history of lower extremity amputation [7].

A descriptive cross-sectional study which has been done using individuals who were having diagnosed diabetic foot ulcers selected from the National Hospital of Sri Lanka in 2011 showed among those patients with foot ulcers, nonhealing ulcers were present among 82.7%, and amputations amounted to 38.2%. Also, they have found that there was a significant association between the foot care knowledge and practices (*p* < 0.001) [8].

According to a cross-sectional study done on diabetic foot: proportion, knowledge, and foot self-care practices among diabetic patients in Dar es Salaam, Tanzania, of 404 patients included in this study, 15% had foot ulcers, 44% had peripheral neuropathy, and 15% had peripheral vascular disease. In multivariate analysis, peripheral neuropathy (*p* < 0.001) and insulin treatment (OR 2.04, 95% CI) were significantly associated with presence of foot ulcer. Patients with moderate peripheral neuropathy were eight times more likely to have diabetic foot ulcer, and those with severe neuropathy were 24 times more likely to develop ulcers. Patients on insulin treatment were twice more likely to have diabetic foot ulcer than those on oral or nonpharmacological management. Foot self-care was significantly higher in patients who had received advice on foot care and in those whose feet had been examined by a doctor at least once [9].

About 85% of the diabetic-related amputations are due to foot ulcers, and this accounts for more than 50% of nontraumatic lower limb amputations [10]. This is at least 15 times greater in those with diabetes than nondiabetics. The American Diabetes Association has estimated that one in five people with diabetes who seek hospital care does so for foot problems [11].

Early proper management of diabetic foot can reduce the severity of complications such as preventable amputations and possible mortality improving overall quality of life. Based on studies, good blood sugar control, wound debridement, proper dressings, and off-loading shoes should always be a part of diabetic foot ulcer management. Furthermore, surgery to heal chronic ulcers and prevent recurrence should be considered an essential component of management. Appropriate patient education encourages good foot care practices and thereby prevents the occurrence of diabetic foot diseases and its complications [12].

The main objective of this descriptive cross-sectional study was to assess proportion, existing knowledge, and practices on diabetic foot among patients attending the diabetic clinic at Colombo South Teaching Hospital which is situated in an urban setting in Sri Lanka. The specific objectives were to assess the proportion of diabetic foot and risk stratification of diabetic patients for diabetic foot diseases, to assess the knowledge and practices on diabetic foot diseases among diabetic patients, and to assess the disease variables and sociodemographic factors associated with diabetic foot disease.

## 2. Methodology and Study Instruments

This is a descriptive cross-sectional study conducted in a diabetic clinic at an urban tertiary care hospital (Colombo South Teaching Hospital) which caters to around 300 patients with diabetes on a daily basis. The referrals to the diabetic clinic are done by the other subspecialties in the hospital itself, patients attending to the outpatients departments, and direct referrals from the primary and secondary care. Even though the patient group attending the diabetic clinic is a representative sample of a Sri Lankan urban population, the sample that attended the foot clinic cannot be considered a representative sample of the Sri Lankan population. In the selected sample, the majority were females (*n* = 245) and most of the patients were above 50 years of age. This may be due to the timing of the clinic which was during the working hours of the day. This may be the reason for getting less males and less people in the 18–50-year age group.

334 patients with diabetes who are above 18 years of age were included into the study by simple random sampling. Exclusion criteria were age of 18 years or less than 18 years, mental subnormality, and inability to comply with medical instructions.

Questionnaires (Appendix 1 in Supplementary Materials (available here)) were developed to get the details about sociodemographic details and disease variables according to the previous studies done on diabetes [8]. Questionnaire to assess knowledge and practices about foot care was adopted from the foot care questionnaire used in the Diabetes Care Program of Nova Scotia [13]. Pretesting of the questionnaire was done before starting the study.

Medical officers in the diabetic clinic of Colombo South Teaching Hospital and a trained research assistant obtained the informed written consent from the patients after checking patient suitability depending on inclusion and exclusion criteria (Appendix 2 in Supplementary Materials).

Questionnaire 3 was translated into 2 other languages which are commonly used in Sri Lanka (Sinhala, Tamil). These were prepared to collect data on sociodemographic factors, disease variables, risk stratification of diabetic foot, and knowledge and practices with regard to diabetic foot. Questionnaires 1 and 2 were interviewer administered which were filled by specially trained medical officers whereas questionnaire 3 was a self-administered questionnaire. Questionnaire 3 was filled by an interviewer if patient has difficulties in filling the form. Questionnaire 2 was administered to collect data on disease variables, medical history, investigations, complications, and diabetic foot with risk stratification. Medical history and investigation findings were taken from the patients' medical records.

Diabetic foot risk assessments were done by trained medical officers by a proper examination of the feet according to risk assessment in the Diabetic Foot Care Program of Nova Scotia. Ankle brachial pressure index (ABPI), monofilament test, and vibration sensation test using a biothesiometer were done using standard techniques.

The monofilament test was done on three sites, and the response recorded. If two out of three responses were incorrect, it was taken as peripheral neuropathy. Vibration sensation of the foot was assessed using a biothesiometer, and anything above 25 Hz was taken as peripheral neuropathy. Ankle brachial pressure index was taken as an objective measure of peripheral vascular disease, using the standard blood pressure cuff and Doppler device to detect pulse. Systolic blood pressure was measured in both arms, and the higher value was used as the denominator of the ABI. Systolic blood pressure is then measured in the dorsalis pedis and posterior tibial arteries by placing the cuff just above the ankle. The higher value is the numerator of the ABI in each limb. A value less than 0.9 was taken as the presence of peripheral vascular disease [14].

At the end of questionnaire 2, patients were categorised into one of the three categories (low, moderate, or high risk for diabetic foot disease). This risk stratification system was developed from the Diabetes Care Program of Nova Scotia [13].

Questionnaire 3 is a self-administered questionnaire which was prepared in all three languages to improve patients' understanding. The objective of this questionnaire was to assess patient's knowledge and practices on foot care, footwear, safety, and prevention of complications with regard to diabetic foot. This questionnaire was a modification of the foot care questionnaire used in the Diabetes Care Program of Nova Scotia. The patients who were having difficulty in answering the questionnaire alone were assisted by an interviewer.

Before the commencement of proper data collection, a pilot study was done using 30 patients to check the reliability of the questionnaires and to check whether the questions were understood by the patients.

### 2.1. Statistical Analysis

SPSS software program (Statistical Package for the Social Sciences) version 20 was used to analyse the data after collection and tabulation. Quantitative data were expressed as mean and SD (standard deviation) while the qualitative data were expressed as numbers and percentages. Chi-squared test was used with 5% level of significance to assess the differences in frequencies of the qualitative variables.

## 3. Results

The mean age of the patients included in the study was 58.23 ± 10.65 years while the median duration of diabetes was 10.54 ± 7.32 years. When the age is categorised into 10-year intervals, majority of the patients were in the 51-60-year age group (37%). Another 29% belonged to the 61-70-year age group.

Among these patients, 245 patients (73.4%) were females and 89 patients (26.6%) were males (Table 1). The mean BMI of the population was 25.38 ± 4.06 kg/m^2^. The mean fasting blood sugar among the group was 135.6 ± 45.5 mg/dl.

Majority of patients have been educated up to the ordinary level (32%) while there were few illiterate people (2.4%). 4.5% of patients have an educational level of degree and above. Fundoscopy findings were available for 66% of the studied population (*n* = 222). 50% of them did not have diabetic retinopathy, and among the patients with diabetic retinopathy, most had nonproliferative diabetic retinopathy (12%). 77.5% of the patients were on oral hypoglycemics, and 4.8% of the patients were using insulin. 17.7% of the patients were using both insulin and oral hypoglycemics. Most of the patients stated that they take their medications regularly (91%).

When considering the macrovascular complications of diabetes mellitus in the studied population (*n* = 333), 14.1% of patients had a history of ischemic heart disease while 85% of them have not had a history of ischemic heart disease. Among 331 patients, 5 patients (1.5%) have given a history of TIA or stroke.

114 patients (34.1%) were having peripheral neuropathy while 97 patients out of 334 (29.5%) were having peripheral vascular disease. However, the diabetic foot disease according to the IWGFDF definition was present only in 50 (15%) patients (Table 2). 78 patients have experienced a foot ulcer which took more than 2 weeks to heal while 254 out of 332 patients have not had experienced a foot ulcer that took more than 2 weeks to heal.

According to the examination findings, 65.3% of patient were in the moderate-risk category while 6% were at high risk and 28.7% were at low risk (Table 3). 6.9% of patients were having diabetic foot disease according to the definition.

When considering the knowledge of the patients on foot care, 58.45% of patients had not read any handouts on foot care while 38.6% of them have never attended a class on diabetic foot care. 61.4% of patients had not read handouts about suitable footwear.

9.4% of patients were using special diabetic footwear while majority (90.6%) were not using special footwear. 46.6% of patients were self-examining their feet daily while 10.5% of them never self-examine their feet. However, 97.3% of patients wash their feet daily and 86.8 patients keep their inter toe areas dry after washing. 48.8% of patients use moisturizer to the feet. 87.4% of patients cut their toenails by themselves while 12% of patients get their family member's help to cut their toenails.

There is a significant association (*p* < 0.05) between the knowledge about foot care and the foot care practices. Also, there is a significant association between current or past foot ulcer which took more than 2 weeks to heal and the knowledge about foot care (*p* < 0.05).

## 4. Discussion

This descriptive cross-sectional study was conducted to assess the knowledge, attitude, and practices of foot care among the diabetic patients attending a diabetic clinic at a tertiary care hospital.

Most of the study population was females, and it was 73.45% (Table 1). The mean age of the patients included in the study was 58.23 ± 10.65 years. A descriptive cross-sectional study which has been done among individuals who were diagnosed with diabetic foot ulcers selected from the National Hospital of Sri Lanka in 2011 showed that the mean age of having diabetic foot ulcers was 58.4 years (SD ±8.6) which is similar to our finding. When the age is categorised into 10-year intervals, majority of the patients were in the 51-60-year age group (37%) (Table 1). The mean BMI of the patients was 25.38 ± 4.06 kg/m^2^ which is in the overweight range of the BMI categorisation for an Asian population. The mean duration of diabetes was 10.5 years. The mean fasting blood glucose level is 135.5 mol/l which showed a poor diabetes control among the population.

Among the study population of 333 patients, 52 (15.2%) patients had an abnormal monofilament test and 108 patients had abnormal vibration sense. Either of these was considered peripheral neuropathy. Depending on these findings, 114 (34.1%) of patients had peripheral neuropathy which is a major risk factor for foot ulceration. A study has published similar results where 44% of patients have had peripheral neuropathy and 15% of patients have had peripheral vascular disease. In their study population, presence of foot ulceration was 15% and they have found a significant association between foot ulceration and presence of peripheral neuropathy and insulin treatment [1].

Our study population was selected from the routine diabetic clinic. This may be the reason for the very low prevalence of diabetic foot disease in the study population. Most of the studies which analysed diabetic foot disease have chosen the study population from the patients already having foot problems [2]. However, our study was mainly designed to assess the proportion and knowledge, attitudes, and practices about foot care; the appropriate population would be the patients attending the general diabetic clinic.

The proportion of patients having diabetic foot according to the IWGDF definition in our study is 50 patients out of 334 (15%) patients. This prevalence is slightly higher than the pooled worldwide prevalence of diabetic foot disease found in a systematic review and meta-analysis done by Zhang et al. [12]. The prevalence of diabetic foot found in this meta-analysis was 6.3%. The definition used in this meta-analysis to define diabetic foot was similar to the WHO definition which we used for our study. A similar prevalence has been stated by Boulton, who reported that the worldwide prevalence of diabetic foot ranges from 1.4% to 5.9%, in his grand overview about diabetic foot [15]. Our prevalence is almost similar when compared to the study done by Chiwanga et al. in the Dar es Salaam, Tanzania [9], who reported the prevalence of diabetic foot ulcer as 15%. However, they have mentioned in their article that the higher prevalence is because they hired most of their patients from diabetic foot clinics. In another study done in Sudan about the prevalence of diabetic foot ulceration and the prevalence of diabetic foot ulcer and associated risk factors, they have found a higher prevalence of diabetic foot ulcer in that study group which was 18.1% [16]. When considering, Sri Lanka, there is only one study done in a diabetic clinic in the National Hospital in Sri Lanka about the prevalence of neuropathic foot ulceration; they have found that the prevalence of neuropathic foot ulceration or amputation due to neuropathic foot ulceration was 10.2% which is slightly less than our finding [7]. The major risk factor for the development of foot ulcers is the presence of peripheral neuropathy according to previous studies. The presence of both peripheral neuropathy and peripheral vascular disease is not essential to develop diabetic foot ulcers. In our study, the number of patients who had current or past foot ulcers which took more than 2 weeks to heal, which is very much suggestive of diabetic foot ulcer, was 78 (23.4%) which is higher than the prevalence of foot ulcer described in Chiwanga et al.'s study in Dar es Salaam, Tanzania [9].

When considering the peripheral vascular disease and peripheral neuropathy separately, the prevalence of peripheral neuropathy was found to be 34.1% and peripheral vascular disease was present in 29.9% of the study population.

Chiwanga et al. [9] have shown a significant association between diabetic foot ulcers and peripheral neuropathy. In our study, we found the similar results. There was a significant association between the peripheral neuropathy and current or past foot ulcers which took more than 2 weeks to heal (*p* < 0.05).

Risk stratification for diabetic foot disease was done according to the criteria used by the Nova Scotia foot risk assessment guide [13]. More than 50% of patients (61.1%) were in the moderate-risk category, and 5.7% of patients were in the high-risk category (Table 2). This is an important guide for the management of diabetic patients. Predicting the risk for foot ulceration early can prevent the limb-threatening complications of diabetic foot disease. This is important because 85% of the amputations in diabetic patients were preceded by a foot ulcer which later gets complicated with infection and gangrene [17]. Therefore, limb amputations and foot ulcerations are significantly interrelated in diabetes and the rate of amputations are 15 times higher among diabetes patients when compared to nondiabetic adults [18].

We assessed the patients' knowledge and practices using a self-administered questionnaire given to the patients in their own language. According to the findings of the questionnaire, 58.4% of patients never had an opportunity to read a handout about diabetic foot care. 38% of the patients have had read a handout about foot care. Our results are slightly higher than the percentage of patients who read handouts about diabetic foot care found in a study done in Makkha, Saudi Arabia, by Goweda et al. which was about 34% [18]. This implies the importance of educating the patients by giving handouts for the patients who are able to read. Most of our study population had a reasonable literacy indicated by the educational level questioned in the interviewer-administered questionnaire. Only 2.4% have never attended a school, and most of them were educated up to the O/L.

61.4% of patients have attended a class about foot care, and this is higher than the percentage found in the study done by Goweda et al. [18]. Regular classes about foot care are a very effective way of educating the diabetic patients about foot care because the patients get a chance to clarify their doubts and the education can be delivered in a customised manner to cater the individual patient's needs. Further, all the newly diagnosed diabetic patients should be educated about foot care on the very first clinic visit and they should be examined for the presence of signs of diabetic foot disease in the very first clinic visit by an experienced person about diabetic foot disease because they may have had diabetes for a long time before they were diagnosed. Moreover, this gives the opportunity to risk stratify them and direct them to appropriate foot care facilities such as correct footwear and foot orthoses. This has a lot of long-term benefits such as reduction of the rate of amputations and long-term disability, reduction of the health care cost for surgeries and different expensive treatment modalities, and improvement of the quality of life of the diabetic patients. According to the literature, every 30s, there is a lower limb amputation due to diabetes [17].

33.1% of patients have read the handouts about diabetic footwear, and this is higher than the percentage found by a study done in Makkha, Saudi Arabia, by Goweda et al. [18]. In our study, only 9.4% of patients were using special footwear for diabetes. 36.8% of patients were using acceptable kinds of footwear such as adjustable shoes, shoes with wide toe space, sandals, athletic shoes, and custom-made shoes. A similar finding was observed in the study done by Goweda et al. in Makkha, Saudi Arabia. 63.2% of patients were using pointed shoes, flip-flops, or high-heeled shoes which are recognised as an improper footwear. Even though we did not find a significant statistical association between the use of appropriate footwear and the diabetic foot disease, only 8 out of 48 patients with diabetic foot disease were using a special diabetic footwear. Further, 65 out of 76 patients with current or past foot ulcer which took more than 2 weeks to heel were not using a special diabetic footwear. An appropriate footwear includes the custom-made footwear according to the patient's foot architecture (for the patients who have existing foot problems such as Charcot foot), off-loading footwear, comfortable and appropriate footwear with wide toe space, and adjustable and well-fitting footwear. An appropriate footwear should be adjustable according to the patient's pressure points in the foot, and it should be made of good-quality material appropriate to the climate of the country. Patients should also be educated about using appropriate footwear according to their activities, and they should be provided with the advices such as suitable socks, how to check the shoe before wearing it, how to identify the faults in the footwear, and what are the precautions to be taken when they are wearing the footwear for a long duration. The major barrier towards the appropriate footwear is the high cost. Custom-made shoes and good-quality shoes are expensive, and they are not freely available in the government hospitals. Even if a patient can afford a pair of good-quality footwear, they do not have the knowledge about choosing it correctly according to their needs. Some patients are reluctant to wear their expensive footwear in adverse weather conditions because they are worrying about the damage to their footwear by the weather. They also cannot afford more than one footwear because of the cost, and they tend to wear the same footwear for all the activities. Sometimes, these footwear are bulky and unsightly which prevent the patient from wearing these frequently. These problems should be addressed adequately to motivate the diabetic patients to wear the appropriate shoes. Sometimes, because of the cultural beliefs and the occupations, patients used to be barefooted. This increases the foot problems related to diabetes, and these were observed in the study conducted by Goweda et al. at Makkha, Saudi Arabia, and Taksande et al. in central rural India [18, 19].

Knowledge about diabetic foot disease was assessed by the self-administered questionnaire. The mean score was 11.28 out of 20 which is slightly higher than 50%. Most of the patients have scored above 10.

When analysing the foot care practices (Table 1), 46.6% of patients were self-examining their feet daily and this is a higher percentage when compared to the study done by Goweda et al. at Makkha, Saudi Arabia. Goweda et al. have found this percentage as 37.7%. 97.3% of patients wash their feet daily, and this is a similar finding to the aforementioned study. However, a study done in Chandigarh, India, has found that only 63.3% of diabetic patients take care of their feet by washing their feet daily [20]. In countries like Saudi Arabia, they wash their feet regularly as a religious ritual which may have accounted for this higher percentage. Our study was conducted in a tertiary care hospital which mainly serves the urban population where the awareness about cleanliness and the sanitation are much better when compared to the above study which was conducted in a rural area in India. 86.8% of patients keep their intertoe areas dry after washing which is a much higher percentage when compared to previous studies [18]. 48.8% of patients use moisturizer to the feet, and 87.4% of patients self-cut their toenails which are much more similar findings to the previous studies [18].

In this current study, there is a significant association (*p* < 0.05) between the knowledge about foot care and the foot care practices. Even though there is no statistically significant association between the presence of diabetic foot and the knowledge about foot care, most of the patients with diabetic foot disease (*n* = 37) have not had any form of knowledge about foot care (reading handouts, attending classes). The similar findings were found in previous studies according to the literature [18]. These findings confirm the importance of educating the patients about diabetic foot disease and correct foot care facilities. Proper education can prevent this limb-threatening complication which has a major impact on health care costs.

## 5. Conclusion

Our study concludes that there is a large group of patients who have a significant risk to develop diabetic foot disease even though the prevalence of diabetic foot disease is 15%. Also, there is a significant lack of knowledge and a high rate of improper foot care practices in this study population. Furthermore, our study shows a significant association between knowledge about foot care and foot care practices and the knowledge about foot care and the development of diabetic foot complications. This confirms the importance of education about foot care as an effective method of primary prevention of diabetic foot complications. Proper education about diabetic foot care reduces the development of diabetic foot disease which has a major impact on the quality of life of a diabetic patient.

## 6. Recommendations

The main recommendation from this study is to improve the patients' knowledge about proper foot care practices. Classes, handouts, posters, and the use of mass media are some effective methods of education. Another recommendation is to increase patients' awareness about correct footwear and increase the availability of custom-made shoes at least for high-risk patients. All the diabetic patients should be examined by a qualified person to detect the features of foot disease, and all should get risk stratified. The patients should be educated according to their risk, and the follow-up plan should be individualised according to the individual patients' risk. Improvement of pediatric services in the diabetic clinics is an essential step to reduce the devastating diabetic foot complications which have a significant impact on health care costs.

## Figures and Tables

**Table 1:** Sociodemographic characteristics of the patients.

Patient characteristics	Frequency	Percentage (%)
Gender		
(i) Female	245	73.4
(ii) Male	89	26.6
Age groups		
(i) 21-30	4	1.2
(ii) 31-40	12	3.6
(iii) 41-50	51	15.3
(iv) 51-60	125	37.4
(v) 61-70	98	29.3
(vi) 71-80	42	12.6
(vii) 81-90	2	0.6
Foot care practice		
Frequency of self-examination of feet		
(i) Everyday	157	47.0
(ii) 2-6 times a week	49	14.7
(iii) Once a week or less	41	12.3
(iv) When there is a problem in feet	48	14.4
(v) Not examining	29	8.7
Washing feet everyday		
(i) Yes	325	97.3
(ii) No	6	1.8
Dry between toes		
(i) Yes	290	86.8
(ii) No	40	12.0
Use of moisturizer for feet		
(i) Yes	163	48.8
(ii) No	166	49.7
Cutting of toenails		
(i) Self	292	87.4
(ii) Family member	40	12.0
Use suitable shoes	112	36.8

**Table 2:** Frequency of diabetic foot complications.

Foot complications		Frequency	Percentage (%)
Current or past foot ulcer which took more than 2 weeks to heal	Yes	78	23.5
	No	254	76.5
Diabetic foot disease	Yes	50	15
	No	284	85

**Table 3:** Risk category of diabetic foot disease.

Risk category	Frequency	Percentage (%)
Low risk	96	28.7
Moderate risk	218	65.3
High risk	20	6

## Data Availability

The observational data used to support the findings of this study are available from the corresponding author upon request.

## Supplementary Materials

Supplementary Materials Study instruments.
