# Supplementary material for: Assessment of Prevalence, Associations ,Knowledge, and Practices about Diabetic Foot Disease in a Tertiary Care Hospital in Colombo, Sri Lanka
Source: Int J Chronic Dis. 2020 Nov 26;2020:4504627. doi: 10.1155/2020/4504627 (PMC7714566; doi:10.1155/2020/4504627)
Supplement: Supplementary Materials — Study instruments. [file 4504627.f1.pdf]

Appendix 1 – Questionnaires

Q 1

Serial number:

CONFIDENTIAL

ANNEXURE 1 - Interviewer administered questionnaire: Sociodemographic data

Prevalence of diabetic foot, existing knowledge and practices on foot care among patients attending the diabetic clinic at Colombo South Teaching Hospital.

i. Age -------- yrs.

ii. Sex: F / M

iii. Address:

iv. Duration of Type 2 Diabetes --------- yrs.

v. Marital status

1. Single		2. Married	
3. Divorced/separated		4. Widowed	
5. Other			

vi. Level of education

1. Not gone to school		2. Grade 1-5	
3. Grade 6-10		4. Up to O/L	
5. Up to A/L		6. Degree and above	

vii. Occupation

1.Manager		2. Professional	
3. Associate professionals/technicians		4. Clerical support workers	
5.Service and sales worker		6.Skilled agricultural, forestry, fishery worker	
7.Craft & related trades worker		8. Plant & machine operators.	
9.Elementry occupation		10.Armed forces	
11.Retired		12.Housewife	
13.Unemployed		14. Other	

viii. Level of monthly income

1.Rs. <10,000		2.Rs. 10,000-20,000	
3.Rs. 20,000- 30,000		4.Rs. 30,000- 40,000	
5.Rs. >40,000			

Serial Number:

Q 2

CONFIDENTIAL

ANNEXURE 2 – Medical history, Diabetes related complications and medication data sheet, to be filled by medical officers.

Prevalence of diabetic foot, existing knowledge and practices on foot care among patients attending the diabetic clinic at Colombo South Teaching Hospital.

Height ---------------cm weight -------------------Kg BMI----------------

Smoking:

Current smoker		Pack years
Ex-smoker		

Alcohol:

Yes		No	

Medical history

Ischaemic heart disease	
Heart failure	
Hypertension	
Dyslipidemia	
PVD	
CVA	
CRF	
Thyroid diseases	
Osteoarthritis	
Osteoporosis	
Bronchial asthma/ COPD	
Cancer	
Connective tissue disorders	
Other - specify	

Investigations		
	Results	Date
**FBS**		
**PPBS**		
**RBS**		
**Hb$_{A1C}$**		
**Lipid Profile:**<br>Total Cholesterol<br>TG<br>HDL<br>LDL<br>Chol/HDL		
**Hb**		

Medication for diabetes

Oral hypoglycemic drugs	
Insulin	

Compliant with medications

1. Yes		2. No	

Complications of Diabetes

Macrovascular complications

	Yes	No
Ischaemic heart diseases		
Stroke/ Transient ischaemic attack		
PVD		

Microvascular complications

Diabetic neuropathy in feet

	R		L	
Abnormal monofilament test	Yes	No	Yes	No
Abnormal vibration sensation	Yes	No	Yes	No

Diabetic retinopathy

No diabetic retinopathy	
Maculopathy	
Non proliferative retinopathy	
Proliferative retinopathy	

Diabetic nephropathy

Presence of albumin in urine (UFR)	Yes/ No
Microalbumin/ creatinine ratio	
Serum Creatinine	
eGFR	

The Diabetic Foot Risk Assessment

❍Low Risk		
**Skin/Nails**		
❍ Dry	R	L
❍ Sweaty	R	L
**Vascular**		
❍ Shiny skin	R	L
❍ Hair Loss	R	L
❍ Edema	R	L

⬣High Risk		
**Skin/Nails**		
⬣ Skin breakdown	R	L
⬣ Ever had a foot ulcer?	Yes	No
Present foot ulcer	R	L
Ulcer within past 1 year	R	L
**Structure**		
⬣ Amputation		
Toe amputations	R	L
Mid tarsal amputations	R	L
Below knee amputations	R	L
Above knee amputations	R	L

▼Moderate Risk		
**Skin/Nails**		
▼ Maceration	R	L
▼ Fissure/cracks	R	L
▼ Corn	R	L
▼ Blister	R	L
▼ Callus	R	L
▼ ↑Temp.	R	L
▼ Thickened nails	R	L
▼ Discolored nails	R	L
▼ Deformed nails	R	L
▼ Ingrown nails	R	L
**Structure**		
▼ Hammer toes / claw toes	R	L
▼ Overlapping digits	R	L
▼ Bunion	R	L
▼ Arch deformity	R	L
**Sensation**		
▼ Diminished	R	L
▼ Absent	R	L
▼ Painful neuropathy	R	L
**Mobility**		
▼ ↓ ROM		
Toes	R	L
Ankle	R	L
**Vascular**		
▼ Edema (weeping)	R	L
▼ Cold skin	R	L
▼ Pallor/cyanosis	R	L
▼ Cap. refill > 3-4 sec	R	L
▼ Absent dorsalis pedis	R	L
▼ Absent posterior tibial	R	L
ABPI	R	L

FOOT CARE/FOOTWEAR

- ○ Poor foot hygiene *(includes long or poorly shaped nails)*
- ○ Needs assistance with foot care *(poor vision, ↓mobility)*
- ○ Inappropriate footwear *(poor style, condition, or fit)*
- ○ No problem noted.

FOOT CARE EDUCATION

- ☐ Foot Care Questionnaire Completed
- ☐ Foot Care Education
- ☐ Foot Care Review
- ☐ Foot Risk Information Sheet Provided

Risk Category	
❍ **Low (Green**	
▼ **Moderate (Yellow)**	
⬣ **High (Red)**	

Diabetic foot

1.	Current or past foot ulcer which took more than 2 weeks to heal	Yes	No
2.	Peripheral vascular disease	Yes	No
3.	Peripheral neuropathy	Yes	No

Presence of all above 3 factors is indicative of presence of diabetic foot.

Diabetic foot	Absent		Present	

Q3

Serial Number:

CONFIDENTIAL

ANNEXURE 3 - Self-administered questionnaire: Diabetic foot care

Prevalence of diabetic foot, existing knowledge and practices on foot care among patients attending the diabetic clinic at Colombo South Teaching Hospital.

History of Foot Problems

- Have you ever had a sore or cut on your foot or leg that took more than two weeks to heal? ❑ Yes ❑ No
- Have you ever had a foot ulcer? ❑ Yes ❑ No
- Have you ever had an amputation of a toe, foot, or leg? ❑ Yes ❑ No

(If yes, date: ____/____/____).

Current Foot or Leg Problems

- Do you have an ulcer, sore, or blister on your feet at this time? ❑ Yes ❑ No
- Do you have blood or discharge on your socks? ❑ Yes ❑ No
- Do you have any calluses on your feet? ❑ Yes ❑ No
- Do you have any numbness, tingling, pins and needles, or itching sensation in your feet? ❑ Yes ❑ No

Foot Care

- Can you reach and **<u>see</u>** the bottoms of your feet? ❑ Yes ❑ No
- Do you examine your feet? (If yes, how often?) ❑ Yes ❑ No

  ❑ Every day ❑ 2-6 times a week

  ❑ Once a week or less ❑ When I have a problem
- Do you wash your feet everyday? ❑ Yes ❑ No

- Do you dry well between the toes? ❑ Yes ❑ No
- Do you use a moisturizing cream on your feet? ❑ Yes ❑ No
- Do you cut your own toenails? (If no, who does this for you?) ❑ Yes ❑ No

❑ Family member ❑ Caregiver ❑ Foot care nurse/ Podiatrist

Foot Wear

What kind of shoes do you wear? (Check all that apply.)

- ❑ pointed toes
- ❑ broad, round toes
- ❑ high heels
- ❑ sandals
- ❑ flip flops
- ❑ athletic/sneakers/runners
- ❑ shoes made of leather or canvas
- ❑ special/custom shoes
- ❑ shoes with adjustable laces, buckles or velcro

Safety and Prevention

Do you ever soak your feet?

❑ Yes ❑ No

Do you always test water temperature before putting your foot in?

❑ Yes ❑ No

Do you use medicated products for warts, corns or calluses?

❑ Yes ❑ No

Do you put moisturizing creams or lotions between your toes?

❑ Yes ❑ No

Do you ever walk around in your bare feet?

❑ Yes ❑ No

Do you ever wear shoes without wearing any socks?

❑ Yes ❑ No

Do you always inspect your shoes for foreign objects or torn linings?

❑ Yes ❑ No

Do you use a hot water bottle or heating pad on your feet?

❑ Yes ❑ No

Do you sit with your legs crossed?

❑ Yes ❑ No

Do you smoke?

❑ Yes ❑ No

Foot Care Education

Have you ever attended a class on how to care for your feet?	❑ Yes	❑ No
Have you ever read any handouts on foot care?	❑ Yes	❑ No
Have you ever read any handouts on proper footwear?	❑ Yes	❑ No
Would you like a handout on how to care for your feet?	❑ Yes	❑ No

Thank you!

Appendix 2 – consent form

Consent Form

Prevalence of diabetic foot, existing knowledge and practices on foot care among patients attending the diabetic clinic at Colombo South Teaching Hospital.

To be completed by the participant.

1. Have you read the information sheet? **Yes/No**
2. Have you received enough information about the study? **Yes/No**
3. Have had an opportunity to discuss this study and ask any questions? **Yes/No**
4. Have you had satisfactory answers to all your questions? **Yes/No**
5. Who explained the study to you? …………………………………………………………………..................
6. Do you understand that you are free to withdraw from the study at any time without having to giving reason and without affecting your future medical care? **Yes/No**
7. Sections of your medical notes and investigation reports will be checked by medical officers/ research assistants. All personal details will be considered as strictly confidential. Do you give permission to access your medical records? **Yes/No**
8. Have you had sufficient time to come to your decision? **Yes/No**
9. Do you agree to take part in this study? **Yes/No**

Name of the participant: ____

Signature: ___________________________

Date: _______________________________

To be completed by the investigator.

I have explained the study to the above participant and he/she has indicated her willingness to take part in this study.

Name of the investigator: ____

Signature: ____________________________

Date: ________________________________
